# Status and the Brain

**DOI:** 10.1371/journal.pbio.1001941

**Published:** 2014-09-02

**Authors:** Amanda V. Utevsky, Michael L. Platt

**Affiliations:** 1 Department of Psychology, Duke University, Durham, North Carolina, United States of America; 2 Center for Cognitive Neuroscience, Duke University, Durham, North Carolina, United States of America; 3 Duke Institute for Brain Sciences, Duke University, Durham, North Carolina, United States of America; 4 Department of Neurobiology, Duke University Medical Center, Durham, North Carolina, United States of America

## Abstract

Social hierarchy is a fact of life for many animals. Navigating social hierarchy requires understanding one's own status relative to others and behaving accordingly, while achieving higher status may call upon cunning and strategic thinking. The neural mechanisms mediating social status have become increasingly well understood in invertebrates and model organisms like fish and mice but until recently have remained more opaque in humans and other primates. In a new study in this issue, Noonan and colleagues explore the neural correlates of social rank in macaques. Using both structural and functional brain imaging, they found neural changes associated with individual monkeys' social status, including alterations in the amygdala, hypothalamus, and brainstem—areas previously implicated in dominance-related behavior in other vertebrates. A separate but related network in the temporal and prefrontal cortex appears to mediate more cognitive aspects of strategic social behavior. These findings begin to delineate the neural circuits that enable us to navigate our own social worlds. A major remaining challenge is identifying how these networks contribute functionally to our social lives, which may open new avenues for developing innovative treatments for social disorders.

## Abstract 2

Status and the Brain A neuroimaging study reveals regions in the monkey brain that track social status—Amanda V. Utevsky and Michael L. Platt put the new findings into context. See accompanying Research Article by MaryAnn P. Noonan, Matthew F. Rushworth, and colleagues.

*“Observing the habitual and almost sacred ‘pecking order’ which prevails among the hens in his poultry yard—hen A pecking hen B, but not being pecked by it, hen B pecking hen C and so forth—the politician will meditate on the Catholic hierarchy and Fascism.” —Aldous Huxley,* Point Counter Point *(1929)*

From the schoolyard to the boardroom, we are all, sometimes painfully, familiar with the pecking order. First documented by the Norwegian zoologist Thorleif Schjelderup-Ebbe in his PhD thesis on social status in chickens in the 1920s, a pecking order is a hierarchical social system in which each individual is ranked in order of dominance [1]. In chickens, the top hen can peck all lower birds, the second-ranking bird can peck all birds ranked below her, and so on. Since it was first coined, the term has become widely applied to any such hierarchical system, from business, to government, to the playground, to the military.

Social hierarchy is a fact of life not only for humans and chickens but also for most highly social, group-living animals. Navigating social hierarchies and achieving dominance often appear to require cunning, intelligence, and strategic social planning. Indeed, the Renaissance Italian politician and writer Niccolo Machiavelli argued in his best-known book “The Prince” that the traits most useful for attaining and holding on to power include manipulation and deception [2]. Since then, the term “Machiavellian” has come to signify a person who deceives and manipulates others for personal advantage and power. 350 years later, Frans de Waal applied the term Machiavellian to social maneuvering by chimpanzees in his book *Chimpanzee Politics* [3]. De Waal argued that chimpanzees, like Renaissance Italian politicians, apply guile, manipulation, strategic alliance formation, and deception to enhance their social status—in this case, not to win fortune and influence but to increase their reproductive success (which is presumably the evolutionary origin of status-seeking in Renaissance Italian politicians as well).

The observation that navigating large, complex social groups in chimpanzees and many other primates seems to require sophisticated cognitive abilities spurred the development of the social brain hypothesis, originally proposed to explain why primates have larger brains for their body size than do other animals [4],[5]. Since its first proposal, the social brain hypothesis has accrued ample evidence endorsing the connections between increased social network complexity, enhanced social cognition, and larger brains. For example, among primates, neorcortex size, adjusted for the size of the brain or body, varies with group size [6],[7], frequency of social play [8], and social learning [9].

Of course, all neuroscientists know that when it comes to brains, size isn't everything [10]. Presumably social cognitive functions required for strategic social behavior are mediated by specific neural circuits. Here, we summarize and discuss several recent discoveries, focusing on an article by Noonan and colleagues in the current issue, which together begin to delineate the specific neural circuits that mediate our ability to navigate our social worlds.

Using structural magnetic resonance imaging (MRI), Bickart and colleagues showed that the size of the amygdala—a brain nucleus important for emotion, vigilance, and rapid behavioral responses—is correlated with social network size in humans [11]. Subsequent studies showed similar relationships for other brain regions implicated in social function, including the orbitofrontal cortex [12] and ventromedial prefrontal cortex [13]. Indeed, one study even found an association between grey matter density in the superior temporal sulcus (STS) and temporal gyrus and an individual's number of Facebook friends [14]. Collectively, these studies suggest that the number and possibly the complexity of relationships one maintains varies with the structural organization of a specific network of brain regions, which are recruited when people perform tests of social cognition such as recognizing faces or inferring others' mental states [15],[16]. These studies, however, do not reveal whether social complexity actively changes these brain areas through plasticity or whether individual differences in the structure of these networks ultimately determines social abilities.

To address this question, Sallet and colleagues experimentally assigned rhesus macaques to social groups of different sizes and then scanned their brains with MRI [17]. The authors found significant positive associations between social network size and morphology in mid-STS, rostral STS, inferior temporal (IT) gyrus, rostral prefrontal cortex (rPFC), temporal pole, and amygdala. The authors also found a different region in rPFC that scaled positively with social rank; as grey matter in this region increased, so did the monkey's rank in the hierarchy. As in the human studies described previously, many of these regions are implicated in various aspects of social cognition and perception [18]. These findings endorse the idea that neural plasticity is engaged in specifically social brain areas in response to the demands of the social environment, changing these areas structurally according to an individual's experiences with others.

Sallet and colleagues also examined spontaneous coactivation among these regions using functional MRI (fMRI). Measures of coactivation are thought to reflect coupling between regions [19],[20]; these measures are observable in many species [21],[22] and vary according to behavior [23],[24], genetics [25], and sex [26], suggesting that coactivation may underlie basic neural function and interaction between brain regions. The authors found that coactivation between the STS and rPFC increased with social network size and that coactivation between IT and rPFC increased with social rank. These findings show that not only do structural changes occur in these regions to meet the demands of the social environment but these structural changes mediate changes in function as well.

One important question raised by the study by Sallet and colleagues is whether changes in the structure and function of social brain areas are specific outcomes of social network size or of dealing with social hierarchy. After all, larger groups offer more opportunity for a larger, more despotic pecking order. In the current volume, Noonan and colleagues address this question directly by examining the structural and functional correlates of social status in macaques independently of social group size [27]. The authors collected MRI scans from rhesus macaques and measured changes in grey matter associated with social dominance. By scanning monkeys of different ranks living in groups of different sizes, the authors were able to cleave the effects of social rank from those of social network size (Figure 1).

**Figure 1:**
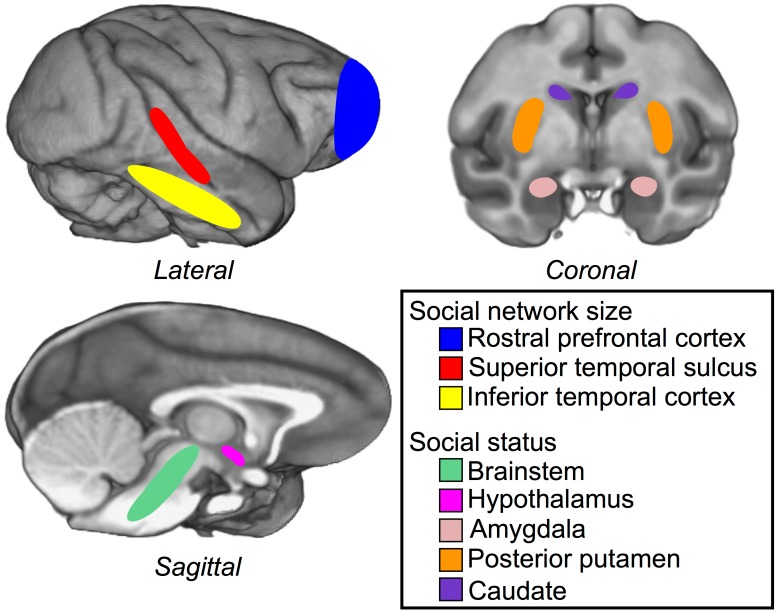
Brain regions in rhesus macaques related to social environment. Primary colors indicate brain regions in which morphometry tracks social network size. Pastel colors indicate brain regions in which morphometry tracks social status in the hierarchy. Regions of interest adapted from [48], overlaid on Montreal Neurological Institute (MNI) macaque template [49].

The authors found a network of regions in which grey matter measures varied with social rank; these regions included the bilateral central amygdala, bilateral brainstem (between the medulla and midbrain, including parts of the raphe nuclei), and hypothalamus, which varied positively with dominance, and regions in the basal ganglia, which varied negatively with social rank. These regions have been implicated in social rank functions across a number of species [28]–[32]. Importantly, these relationships were unique to social status. There was no relationship between grey matter in these subcortical areas and social network size, endorsing a specific role in social dominance-related behavior. Nevertheless, grey matter in bilateral mid-STS and rPFC varied with both social rank and social network size, as reported previously. These findings demonstrate that specific brain areas uniquely mediate functions related to social hierarchy, whereas others may subserve more general social cognitive processes.

Noonan and colleagues next probed spontaneous coactivation using fMRI to examine whether functional coupling between any of these regions varied with social status. They found that the more subordinate an animal, the stronger the functional coupling between multiple regions related to dominance. These results suggest that individual differences in social status are functionally observable in the brain even while the animal is at rest and not engaged in social behavior. These findings suggest that structural changes associated with individual differences in social status alter baseline brain function, consistent with the idea that the default mode of the brain is social [33] and that the sense of self and perhaps even awareness emerge from inwardly directed social reasoning [34].

These findings resonate with previous work on the neural basis of social dominance in other vertebrates. In humans, for example, activity in the amygdala tracks knowledge of social hierarchy [28],[35] and, further, shows activity patterns that uniquely encode social rank and predict relevant behaviors [28]. Moreover, recent research has identified a specific region in the mouse hypothalamus, aptly named the “hypothalamic attack area” [36],[37]. Stimulating neurons in this area immediately triggers attacks on other mice and even an inflated rubber glove, while inactivating these neurons suppresses aggression [38]. In the African cichlid fish *Haplochromis burtoni*, a change in the social status of an individual male induces a reversible change in the abundance of specialized neurons in the hypothalamus that communicate hormonally with the pituitary and gonads [39]. Injections of this hormone in male birds after an aggressive territorial encounter amplifies the normal subsequent rise in testosterone [40]. Serotonin neurons in the raphe area of the brainstem also contribute to dominance-related behaviors in fish [29],[31] and aggression in monkeys [41].

Despite these advances, there are still gaps in our understanding of how these circuits mediate status-related behaviors. Though regions in the amygdala, brainstem, and hypothalamus vary structurally and functionally with social rank, it remains unknown precisely how they contribute to or respond to social status. For example, though amygdala function and structure correlates with social status in both humans and nonhuman primates [27],[28],[35],[42], it remains unknown which aspects of dominance this region contributes to or underlies. One model suggests that the amygdala contributes to learning or representing one's own status within a social hierarchy [28],[35]. Alternatively, the amygdala could contribute to behaviors that support social hierarchy, including gaze following [43] and theory of mind [44]. Lastly, the amygdala could contribute to social rank via interpersonal behaviors or personality traits, such as aggression [45], grooming [45], or fear responses [46],[47]. Future work will be critical to determine how signals in these regions relate to social status; direct manipulation of these regions, possibly via microstimulation, larger-scale brain stimulation (e.g., transcranial magnetic stimulation and transcranial direct current stimulation), or temporary lesions, will be critical to better understand these relationships.

The work by Noonan and colleagues suggests new avenues for exploring how the brain both responds to and makes possible social hierarchy in nonhuman primates and humans. The fact that the neural circuits mediating dominance and social networking behavior can be identified and measured from structural and functional brain scans even at rest suggests the possibility that similar measures can be made in humans. Although social status is much more complex in people than it is in monkeys or fish, it is just as critical for us and most likely depends on shared neural circuits. Understanding how these circuits work, how they develop, and how they respond to the local social environment may help us to understand and ultimately treat disorders, like autism, social anxiety, or psychopathy, that are characterized by impaired social behavior and cognition.
